# ASO Author Reflections: Contemporary Redlining—Direct and Mediating Effects on Healthcare Disparities Among Patients with Gastrointestinal Cancer

**DOI:** 10.1245/s10434-024-16417-z

**Published:** 2024-11-04

**Authors:** Odysseas P. Chatzipanagiotou, Timothy M. Pawlik

**Affiliations:** Department of Surgery, The Ohio State University, Columbus, OH USA

## Past

The term “redlining” originated in the 1930s from color-coded maps used by the Home Owner’s Loan Corporation (HOLC) and the Federal Housing Administration (FHA), which ranked neighborhoods based on risk for investment and mortgage lending. The lowest grade “D” or “hazardous” was commonly assigned to working-class and minority neighborhoods, especially with Black and immigrant populations, and red lines were drawn to outline these areas.^1^ As such, people residing in these neighborhoods were systematically denied loans and economic opportunities, thus perpetuating the problem of marginalization and economic disinvestment. Previous studies have mostly focused on redlining alone, failing to deconstruct and comprehend the complex causal pathways that link redlining with healthcare disparities. Understanding the association between modern day redlining and healthcare disparities requires accounting for the influence of multiple factors that form a complex ecosystem leading to marginalized patients presenting with more comorbidities or advanced disease, as well as receiving inequitable healthcare.^2^ Therefore, the goal of the current study was to characterize the effect of redlining as a root cause of healthcare inequalities, ranging from healthcare access to utilization of health resources, as well as clinical outcomes among patients with GI cancer. Of note, demographic, socioeconomic, and neighborhood factors were assessed at the census-tract level via mediation analysis, which allowed for the definition and quantification of their contributing effect along the causal healthcare pathway.

## Present

Among 94,988 patients with gastrointestinal cancer, 32.2% resided in high (*n*=23, 872) and highest (*n*=6, 791) redlining census tracts versus 46.2% (*n*=43,863) in neutral and 21.6% (*n*=20, 472) in low redlining tracts. The proportion of Black, Hispanic, and White patients experiencing high and highest redlining was 65.9% (*n*=5, 340), 41.6% (*n*=3,134), and 27.9% (*n*=19, 972), respectively. Of note, the current study demonstrated that although redlining directly impacted mortality (23.6%), the majority of the observed association was driven by indirect effects, including advanced disease stage at diagnosis (11.8%), non-receipt of stage-appropriate treatment (48.4%), and CCI (16.2%). Focusing on these indirect associations, residing in areas with highest redlining was associated with 18.2% higher odds of advanced disease at diagnosis. Furthermore, patients from the highest redlining areas had 36.3% greater odds of not undergoing surgery for localized disease (aOR 1.363, 95%CI 1.219–1.524), as well as 38.5% higher odds of not receiving chemotherapy for advanced disease (aOR 1.385, 95%CI 1.216–1.577). Interestingly, the mediation models demonstrated that socioeconomic status [Relative Risk (RR) 1.84, 95%CI 1.11–2.58] mediated 45.6% of the association between redlining and non-receipt of surgery for localized disease, while HPSA status (RR 1.31, 95%CI 1.11–1.53) and racial/ethnic minority status (RR 1.30, 95%CI 1.11–1.49) contributed 20.5% and 19.7% of the effect, respectively. Housing & transportation had a smaller effect (RR 1.21, 95%CI 1.11–2.58) accounting for 14.2% of the total association between redlining and receipt of stage-appropriate surgery. Moreover, the association between redlining and non-receipt of chemotherapy for advanced disease was mediated 37.9 and 33.3% by ICE (RR 1.76, 95%CI 1.20–2.32) and socioeconomic status (RR 1.64, 95%CI 1.01–2.28), respectively. HPSA status was responsible for 24% of the relative increase in the risk of patients residing in redlined areas not receiving chemotherapy for advanced disease (RR 1.24, 95%CI 1.08–1.40); 14.3% of the association was mediated by housing & transportation (RR 1.24, 95%CI 1.07–1.41) (all *p* < 0.05).

## Future

The findings of the current study reveal the intricate web and dynamic interaction of socioeconomic characteristics inside neighborhoods, clinical factors and redlining. ^2^ Contemporary redlining was associated with disparities in equal access to healthcare, health resource utilization, and clinical outcomes among patients with GI cancer. A large proportion of inequities was mediated by clinical, demographic, socioeconomic, and structural factors faced by patients residing in redlined neighborhoods. Understanding the impact of these contextual effects may help identify targets for public health policies and multilevel interventions.^3^ At the neighborhood level, coordinated efforts should focus on reinvesting and revitalizing segregated communities, aiming to improve educational and employment opportunities, good-quality infrastructure, social services, and equal access to care.^4^ To this end, establishment of outreach clinics in segregated areas, development of transportation infrastructure, and systematic identification of social needs in the perioperative setting could contribute significantly to improving healthcare access, clinical outcomes, and overall quality of healthcare.^5^ Rather than a historic remnant of discriminatory policies, contemporary redlining should be addressed to ensure equitable cancer care for all.
